# Comparison of the cachexia index based on hand-grip strength (H-CXI) with the original CXI for the prediction of cancer cachexia and prognosis in patients who underwent radical colectomy for colorectal cancer

**DOI:** 10.3389/fnut.2024.1290299

**Published:** 2024-02-20

**Authors:** Xia-Lin Yan, Lian-Ming Wu, Xiu-Bo Tang, Zong-Ze Li, Zhao Zhang, Hao-Jie Jiang, Zhang-Tao Chen, Ding-Hao Chen, Jiang-Yuan Li, Xian Shen, Dong-Dong Huang

**Affiliations:** ^1^Department of Colorectal Anal Surgery, The First Affiliated Hospital of Wenzhou Medical University, Wenzhou, China; ^2^Department of General Surgery, Yuhuan Second People 's Hospital, Taizhou, China; ^3^School of Clinical Medicine, Wenzhou Medical University, Wenzhou, China; ^4^Department of Gastrointestinal Surgery, The First Affiliated Hospital of Wenzhou Medical University, Wenzhou, China; ^5^Radiology Imaging Center, The First Affiliated Hospital of Wenzhou Medical University, Wenzhou, China

**Keywords:** cancer cachexia, cachexia index, muscle mass, hand-grip strength, colorectal cancer, prognosis

## Abstract

**Background and aims:**

The cachexia index (CXI) is a novel biomarker for estimating cancer cachexia. The cachexia index based on hand-grip strength (H-CXI) has been recently developed as a simple proxy for CXI. The present study aims to compare both the H-CXI and CXI for the prediction of cancer cachexia and postoperative outcomes in patients who underwent radical colectomy for colorectal cancer.

**Methods:**

Patients who underwent radical operations for colorectal cancer were included in this study. Cancer cachexia was diagnosed according to the international consensus outlined by Fearon et al. The cachexia index (CXI) was calculated as [skeletal muscle index (SMI) × serum albumin/neutrophil-to-lymphocyte ratio (NLR)]. The H-CXI was calculated as [hand-grip strength (HGS)/height^2^ × serum albumin/NLR]. The SMI was measured based on the preoperative CT images at the third lumbar vertebra (L3) level. HGS was measured before surgery.

**Results:**

From July 2014 to May 2021, a total of 1,411 patients were included in the present study, of whom 361 (25.6%) were identified as having cancer cachexia. Patients with cachexia had a lower CXI (*p* < 0.001) and lower H-CXI (*p* < 0.001) than those without cachexia. A low CXI but not low H-CXI independently predicted cancer cachexia in the multivariate analysis (OR 1.448, *p* = 0.024). Both a low CXI (HR 1.476, *p* < 0.001 for OS; HR 1.611, *p* < 0.001 for DFS) and low H-CXI (HR 1.369, *p* = 0.007 for OS; HR 1.642, *p* < 0.001 for DFS) were independent predictors for overall survival (OS) and disease-free survival (DFS) after adjusting for the same covariates. A low H-CXI but not low CXI was an independent risk factor for postoperative complications (OR 1.337, *p* = 0.044). No significant association was found between cancer cachexia and postoperative complications.

**Conclusion:**

The CXI and H-CXI exhibited better prognostic value than cancer cachexia for the prediction of postoperative outcomes in patients who underwent radical colectomy for colorectal cancer. The H-CXI was a superior index over the CXI in predicting short-term clinical outcomes, whereas the CXI demonstrated a closer correlation with Fearon’s criteria for cancer cachexia. Ideal tools for the assessment of cancer cachexia should incorporate not only weight loss but also muscle mass, physical function, and inflammatory state.

## 1 Introduction

Cancer cachexia is a multifactorial syndrome characterized by the ongoing loss of skeletal muscle mass that cannot be fully reversed by conventional nutritional support (1). Cancer cachexia is associated with impaired physical function (2), more treatment-related toxicity (3), and reduced survival (4). As indicated by a review from Haehling et al., cancer cachexia is believed to be the direct cause of mortality in more than 30% of patients with cancer, and more than 50% of patients with cancer may have died with cachexia being present (5). Colorectal cancer is the third most common malignancy and ranks second in cancer mortality worldwide (6). Cancer cachexia was detected in 55% of patients with stage IV colorectal cancer and was significantly associated with worse overall survival (7). Therefore, the early identification and management of cancer cachexia are significant, which could provide a potential strategy to improve the prognosis of patients with colorectal cancer.

The diagnostic criteria for cancer cachexia varied in the literature (1, 8, 9). In 2011, the international consensus for the definition of cancer cachexia was published by Fearon et al., and it has become the most accepted criteria so far (1). Weight loss was the most important element in the diagnosis of cancer cachexia shared by these criteria. However, several previous studies have found that cancer cachexia diagnosed by weight loss is not an optimal index for the prediction of clinical outcomes (7, 10). Cancer cachexia diagnosed by the Fearon criteria has been reported to have a low agreement with the clinical presentation of cachexia in patients with colorectal cancer (11). Reduced muscle mass and function and increased systemic inflammation are also significant characteristics of cancer cachexia. Therefore, new tools incorporating muscle mass/function and inflammatory indices are needed for the better monitoring of cachexia and prognosis in patients with cancer.

The cachexia index (CXI) was originally developed by Jafri et al. in 2015 to assess the degree of cachexia in patients with advanced non-small-cell lung cancer (NSCLC) (12). The CXI is an objective index calculated as skeletal muscle index (SMI) × serum albumin/neutrophil lymphocyte ratio (NLR). Jafri et al. reported that patients with a low CXI exhibited worse overall survival and progression-free survival (12). Subsequently, several studies from other groups demonstrated that CXI was significantly associated with prognosis in patients with malignancies, such as lung cancer (13) and lymphomas (14). A recent study showed that the CXI was better than the Fearon criteria for cancer cachexia in predicting overall survival in patients with colorectal cancer (15).

However, the measurement of the SMI is complex, and abdominal CT scans are not routinely performed for many types of malignancies, which impedes the clinical application of the CXI. In 2022, Xie et al. developed a hand-grip strength (HGS)-based cancer cachexia index (H-CXI) as a potential predictor of cancer cachexia and prognosis in patients with cancer. The H-CXI was calculated as [HGS (kg)/height (m)^2^ × serum albumin (g/L)]/NLR. The authors included a nationwide cohort of 14,682 patients with cancer from 41 Chinese medical institutions and found that a low H-CXI was an independent risk factor predicting adverse short-term outcomes and cancer cachexia in patients with cancer (16). The H-CXI is a simple index that has an advantage over the CXI in clinical applications. However, no evidence proves the superiority of the H-CXI over CXI in terms of the prognostic value for clinical outcomes, which is partially due to the lack of high-quality studies investigating the two indices. The present study aims to compare the H-CXI and CXI for the prediction of cancer cachexia and postoperative outcomes in patients who underwent radical colectomy for colorectal cancer.

## 2 Materials and methods

### 2.1 Patients

Patients who underwent radical operations for colorectal cancer at the Gastrointestinal Surgical Department, The First Affiliated Hospital of Wenzhou Medical University, China, were included in this study from July 2014 to May 2021. This study included patients who (1) were ≥ 18 years old, (2) had an American Society of Anesthesiologists (ASA) grade not more than III, (3) planned to receive operations for colorectal cancer with curative intent, (4) had abdominal computed tomography (CT) scans available for review within 1 month before surgery, (5) had a blood routine examination within 1 week before surgery, and (6) agreed to take part in the study and sign an informed consent form. Patients with situations of emergency, such as acute abdomen, intestinal obstruction, and acute inflammation, were not included in the present study. We excluded patients who were unable to be measured for hand-grip strength before surgery and those who were confirmed with cancer metastasis during surgery or underwent palliative surgery. All patients were informed that their clinical information would be used anonymously for research purposes and signed a consent form. This study protocol has been approved by the ethics review board of the First Affiliated Hospital of Wenzhou Medical University. The present study was part of a large-scale prospective study registered in the China Clinical Trial Registry (No. ChiCTR1800019717).

### 2.2 Data collection

The following data were prospectively collected by specialized investigators: (1) preoperative patient demographic and clinical features, including age, sex, body mass index (BMI), Nutritional Risk Screening 2002 (NRS2002) scores, an American Society of Anesthesiologists (ASA) grade, comorbidity, a previous abdominal operation, tumor location, hemoglobin and serum albumin concentration, and neutrophil and lymphocyte counts; (2) operative details, including laparoscopic surgery and combined organ resection; (3) postoperative pathological characteristics of tumor, including histopathology differentiation and tumor-node-metastasis (TNM) stage; (4) short-term postoperative outcomes, including postoperative complications within 30 days of surgery, length of postoperative hospital stay, and costs during hospitalization; and (5) long-term survival obtained by a follow-up after surgery. Postoperative complications classified as grade II or above according to the Clavien–Dindo classification were analyzed (17).

### 2.3 Diagnosis of cancer cachexia and the calculation of the cachexia index

Patients who met one of the three following criteria were diagnosed with cancer cachexia according to the international consensus by Fearon et al. (1): (1) weight loss >5% over the past 6 months; (2) BMI <20 and any degree of weight loss >2%; or (3) low skeletal muscle index (SMI) and any degree of weight loss >2%. A cutoff value for low SMI was referenced from the study by Zhuang et al. (18) based on the Chinese population, which was 34.9 cm^2^/m^2^ for women and 40.8 cm^2^/m^2^ for men. The cachexia index (CXI) was calculated based on the SMI (12) or hand-grip strength (HGS) (16) and was referred to as the CXI or H-CXI, respectively. The CXI was calculated as SMI (cm^2^/m^2^) × serum albumin (g/L)/neutrophil-to-lymphocyte ratio (NLR) (12). The H-CXI was calculated as HGS (kg)/height (m)^2^ × serum albumin (g/L)/NLR (16). The cutoff values for a low CXI and H-CXI were defined using the sex-specific lower quartile, which were 483.25 for the CXI and 98.51 for the H-CXI in males, and 372.96 for the CXI and 66.32 for the H-CXI in females. To measure the SMI, CT images at the third lumbar vertebra (L3) level were analyzed using the image processing system (version 3.0.11.3 BN17 32 bit; INFINITT Healthcare Co., Ltd). To reduce the bias in the measurement of muscle mass, one specialized investigator (Z-Z Li) was trained to analyze the muscle mass under the supervision of an experienced radiologist (Z Zhang). Representative CT images of patients with and without skeletal muscle atrophy are shown in Supplementary Figure S1. Skeletal muscle was identified by Hounsfield unit (HU) thresholds within a range from −29 to +150 and normalized for height (m^2^) to calculate the SMI. HGS was measured using electronic hand-grip dynamometers (EH101, Camry, China) before surgery. Patients were guided to grip the hand-grip dynamometers with the dominant hand with all their strength. The maximum value from three consecutive tests was recorded.

### 2.4 Follow-up

All patients were followed up 1 month after surgery, then every 3 months for the first 2 years, and every 6 months thereafter. The patients were kept in contact with specialized investigators through telephone calls. The patients were scheduled for outpatient visits on the dates of their follow-ups. The follow-up programs included physical examinations, laboratory tests, and radiological examinations such as CT, ultrasonography, and endoscopy as needed. The last follow-up date was January 2022. Overall survival (OS) was calculated from the date of surgery to the date of death from any cause. Disease-free survival (DFS) was calculated from the date of surgery to the date of cancer recurrence or death from any cause, whichever came first.

### 2.5 Statistical analysis

Continuous data were presented as medians with interquartile ranges (IQR). Categorical variables were presented as numbers and percentages. Differences between groups were analyzed using the Mann–Whitney U test or Kruskal–Wallis test for continuous variables and Pearson’s chi-squared tests or Fisher’s exact tests for categorical variables. Survival curves were estimated using the Kaplan–Meier method. Log-rank tests were used to test the difference between the groups for the survival data. Logistic regression analysis was conducted to identify risk factors for postoperative complications and cancer cachexia. Cox proportional hazards models were constructed to identify risk factors for OS and DFS. Variables that were considered clinically relevant and candidate variables with P of <0.1 in the univariate analysis were entered into multivariate analysis to identify independent risk factors. We conducted two separate multivariable analyses for complications, OS, DFS, and cancer cachexia including a low CXI or H-CXI, respectively. The cutoff values for the CXI and H-CXI were defined based on the sex-specific lower quartile. All variables were examined for their multicollinearity before inclusion in the multivariate analyses by calculating the variance inflation factor (VIF). Factors with variance inflation factors (VIF) ≥10 were excluded from the multivariate analysis (19). *Post-hoc* analysis for the sample size and power of the study was conducted based on postoperative complications as the main event using PASS software version 11.0. Based on our sample size, the power for a chi-squared test was 0.837 for a low CXI and 0.962 for a low H-CXI. The data were analyzed using SPSS statistics version 22.0 (IBM, United States) and Empower Stats software (version 2.0). *p*-values of <0.05 were considered statistically significant.

## 3 Results

### 3.1 Baseline characteristics

A total of 1,411 patients were included in the study, including 539 women and 872 men. The median age of the patients was 66 years. Based on the sex-specific lower quartile of the cachexia index, there were 353 patients who were classified as low CXI and low H-CXI groups. Specifically, 255 patients had both a low CXI and low H-CXI, 98 patients had only a low CXI but not a low H-CXI, and 98 patients had only a low H-CXI but not a low CXI. The CXI and H-CXI showed a good agreement (kappa = 0.822, *p* < 0.001). Patients with a low CXI or low H-CXI were older, had a lower BMI, SMI, and HGS, lower levels of albumin and hemoglobin and higher NLR, and had a higher incidence of nutritional risk (NRS2002 ≥ 3) than patients with a high CXI or high H-CXI (Table 1).

**Table 1:** Baseline characteristics of the patients.

Characteristics	All (*n* = 1,411)	High CXI (*n* = 1,058)	Low CXI (*n* = 353)	*p*	High H-CXI (*n* = 1,058)	Low H-CXI (*n* = 353)	*p*
Age, median (IQR), years	66 (7)	64 (16)	69 (16)	<0.001^*^	64 (16)	71 (15)^*^	<0.001^*^
Sex				0.984			0.984
Female	539 (38.2)	404 (38.2)	135 (38.2)		404 (38.2)	135 (38.2)	
Male	872 (61.8)	654 (61.8)	218 (61.8)		654 (61.8)	218 (61.8)	
BMI, median (IQR), kg/m^2^	22.7 (4.2)	22.8 (4.3)	22.2 (4.0)	<0.001^*^	22.8 (4.3)	22.2 (3.8)	<0.001^*^
Weight loss							
<2%	955 (67.7)	719 (68.0)	236 (66.9)	0.701	715 (67.6)	240 (68.0)	0.887
≥2%	456 (32.3)	339 (32.0)	117 (33.1)		343 (32.4)	113 (32.0)	
Albumin, median (IQR), g/L	38.1 (5.8)	38.4 (5.5)	36.3 (6.4)	<0.001^*^	38.6 (5.4)	36.1 (6.1)	<0.001^*^
Hemoglobin, median (IQR), g/L	124 (31)	127 (30)	117 (30.5)	<0.001^*^	127 (29)	116 (30.5)	<0.001^*^
NLR, median (IQR)	2.49 (1.75)	2.16 (1.08)	4.52 (2.49)	<0.001^*^	2.20 (1.19)	4.33 (2.94)	<0.001^*^
NRS2002 scores				0.043^*^			0.001^*^
<3	980 (69.5)	750 (70.9)	230 (65.2)		759 (71.7)	221 (62.6)	
≥3	431 (30.5)	308 (29.1)	123 (34.8)		299 (28.3)	132 (37.4)	
ASA grade				0.714			0.052
I	393 (27.8)	296 (28.0)	97 (27.5)		301 (28.4)	92 (26.1)	
II	863 (61.2)	642 (60.7)	221 (62.6)		653 (61.7)	210 (59.5)	
III	155 (11.0)	120 (11.3)	35 (9.9)		104 (9.8)	51 (14.4)	
Previous abdominal surgery				0.810			0.489
No	1,137 (80.6)	851 (80.4)	286 (81.0)		857 (81.0)	280 (79.3)	
Yes	274 (19.4)	207 (19.6)	67 (19.0)		201 (19.0)	73 (20.7)	
SMI, median (IQR), cm^2^/m^2^	43.2 (13.2)	44.5 (13.2)	39.9 (10.5)	<0.001^*^	43.9 (13.3)	41.2 (11.9)	<0.001^*^
HGS, median (IQR), kg	25.1 (13.7)	25.9 (13.2)	23.2 (13.5)	<0.001^*^	27.4 (12.8)	18.3 (13.2)	<0.001^*^
Tumor location				0.909			0.384
Colon	863 (61.2)	648 (61.2)	215 (60.9)		654 (61.8)	209 (59.2)	
Rectum	548 (38.8)	410 (38.8)	138 (39.1)		404 (38.2)	144 (40.8)	
Differentiation of tumor				0.538			0.437
Poorly differentiated	206 (14.6)	158 (38.8)	48 (13.6)		150 (14.2)	56 (15.9)	
Well differentiated	1,205 (85.4)	900 (61.2)	305 (86.4)		908 (85.8)	297 (84.1)	
TNM stage				0.375			0.171
I	351 (24.9)	273 (25.8)	78 (22.1)		276 (26.1)	75 (21.3)	
II	571 (40.5)	422 (39.9)	149 (42.2)		418 (39.5)	153 (43.3)	
III	489 (34.6)	363 (34.3)	126 (35.7)		364 (34.4)	125 (35.4)	
Laparoscopy-assisted surgery				0.246			0.017^*^
No	630 (44.6)	463 (43.8)	167 (47.3)		453 (42.8)	177 (50.1)	
Yes	781 (55.4)	595 (56.2)	186 (52.7)		605 (57.2)	176 (49.9)	
Combined organ resection				0.601			0.798
No	1,327 (94.0)	993 (93.9)	334 (94.6)		996 (94.1)	331 (93.8)	
Yes	84 (6.0)	65 (6.1)	19 (5.4)		62 (5.9)	22 (6.2)	
Follow-up time, median (IQR), months	50.1 (6.4)	50.3 (6.9)	48.3 (14.1)	0.298	50.3 (7.0)	48.3 (13.8)	0.825

CXI, cachexia index; IQR, interquartile range; BMI, body mass index; NLR, neutrophil-to-lymphocyte ratio; NRS2002, Nutritional Risk Screening 2002; ASA, American Society of Anesthesiologists; SMI, skeletal muscle index; HGS, hand-grip strength; TNM, tumor-node-metastasis. The values in the table were number of patients and percentage unless indicated otherwise. ^*^Statistically significant.

### 3.2 Short-term postoperative outcomes

Of the 1,411 patients, postoperative complications occurred in 347 (24.6%) of them. Both a low CXI and low H-CXI showed a significant correlation with a higher incidence of postoperative complications. Details of short-term postoperative outcomes are listed in Supplementary Table S1. Patients with a low H-CXI had significantly longer postoperative hospital stays and more costs than those with a high H-CXI. However, no significant association was found between a low CXI and length of postoperative hospital stay or costs. The univariate analysis showed that a low CXI, a low H-CXI, age ≥ 75 years, ASA grade III, and TNM stage III were associated with the occurrence of postoperative complications, whereas laparoscopic surgery was negatively associated with postoperative complications. The multivariate analysis showed that age ≥ 75 years (*p* < 0.001) and ASA grade III (*p* = 0.010) were independent risk factors for postoperative complications, whereas laparoscopic surgery was an independent protective factor (*p* = 0.032). Notably, when a low H-CXI was included in the multivariate model instead of a low CXI, a low H-CXI was identified as a significant risk factor for postoperative complications in the multivariate analysis (*p* = 0.044) after adjusting for the same covariates (Table 2). Moreover, a low H-CXI showed better sensitivity (32.3% vs. 30.5%), specificity (77.3% vs. 76.8%), accuracy (66.5% vs. 65.4%), positive predictive value (PPV, 31.7% vs. 30.0%), negative predictive value (NPV, 77.8% vs. 77.2%), and larger area under the ROC curve (AUC, 0.548 vs. 0.537) in the prediction of postoperative complications (Table 3).

**Table 2:** Univariate and multivariate analyses of factors associated with postoperative complications.

Factors	Univariate analysis		Multivariate analysis			
			Low CXI		Low H-CXI	
	OR (95%CI)	*p*	OR (95%CI)	*p*	OR (95%CI)	*p*
Age, years						
≥75/<75	2.298 (1.755–3.009)	**< 0.001** ^*^	2.054 (1.548–2.724)	**< 0.001** ^*^	2.009 (1.510–2.673)	**< 0.001** ^*^
Sex						
Male/Female	1.042 (0.812–1.338)	0.745	1.056 (0.817–1.365)	0.676	1.055 (0.816–1.363)	0.683
BMI, kg/m^2^						
≥25/<25	1.209 (0.914–1.599)	0.184	–	–	–	–
Low CXI						
Yes/No	1.455 (1.112–1.904)	**0.006** ^*^	1.298 (0.981–1.718)	0.068		
Low H-CXI						
Yes/No	1.628 (1.246–2.125)	**< 0.001** ^*^	–	–	1.337 (1.008–1.772)	**0.044** ^*^
Cachexia						
Yes/No	1.199 (0.913–1.574)	0.192	–	–	–	–
Anemia						
Yes/No	1.237 (0.962–1.590)	0.098	0.955 (0.730–1.250)	0.739	0.950 (0.725–1.243)	0.707
NRS2002						
≥3/<3	1.074 (0.827–1.395)	0.591	–	–	–	–
ASA grade						
III/I, II	1.876 (1.318–2.670)	**< 0.001** ^*^	1.618 (1.120–2.337)	**0.010** ^*^	1.575 (1.092–2.274)	**0.015** ^*^
Previous abdominal surgery						
Yes/No	0.943 (0.692–1.285)	0.710	–	–	–	–
Tumor location						
Rectum/Colon	1.070 (0.835–1.371)	0.591	–	–	–	–
Differentiation of tumor						
Low/median or high	1.314 (0.946–1.826)	0.103	–	–	–	–
TNM stage						
II/<I	1.307 (0.948–1.801)	0.102	1.200 (0.863–1.669)	0.279	1.198 (0.861–1.666)	0.284
III/<I	1.418 (1.022–1.967)	**0.037** ^*^	1.324 (0.946–1.854)	0.101	1.325 (0.946–1.854)	0.101
Combined organ resection						
Yes/No	1.243 (0.762–2.029)	0.383	–	–	–	–
Laparoscopic surgery						
Yes/No	0.691 (0.541–0.881)	**0.003** ^*^	0.758 (0.590–0.974)	**0.030** ^*^	0.761 (0.592–0.977)	**0.032** ^*^

OR, odds ratio; CI, confidence interval; BMI, body mass index; CXI, cachexia index; NRS2002, Nutritional Risk Screening 2002; ASA, American Society of Anesthesiologists; TNM, tumor-node-metastasis. ^*^ Statistically significant.

**Table 3:** Sensitivity and specificity of a low CXI and low H-CXI for the prediction of postoperative complications.

Factors	Sensitivity (%)	Specificity (%)	Accuracy (%)	PPV (%)	NPV (%)	AUC, 95% CI
Low CXI	30.5	76.8	65.4	30.0	77.2	0.537 (0.501–0.572)
Low H-CXI	32.3	77.3	66.5	31.7	77.8	0.548 (0.513–0.584)

PPV, positive predictive value; NPV, negative predictive value, AUC, area under the ROC curve. Postoperative complications classified as Grade II or above by the Clavien–Dindo classification were analyzed.

### 3.3 Long-term survival

**Figure 1:**
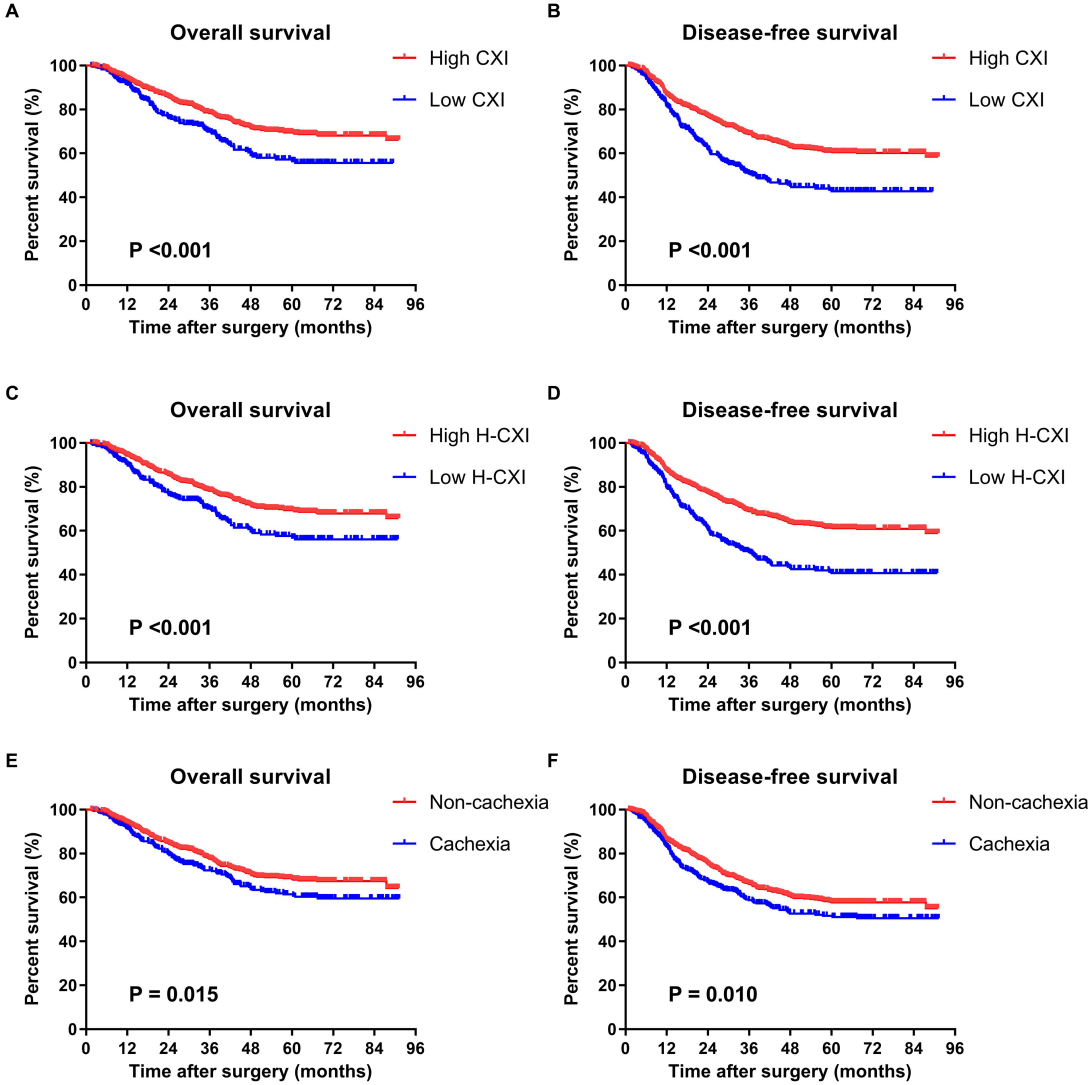
Kaplan–Meier survival curves for associations between survival and the CXI, H-CXI, or cancer cachexia. **(A)** Overall survival and **(B)** disease-free survival of patients with a high CXI and low CXI; **(C)** overall survival and **(D)** disease-free survival of patients with a high H-CXI and low H-CXI; and **(E)** overall survival and **(F)** disease-free survival of patients with and without cancer cachexia.

The median follow-up period was 50.1 months. Among 1,411 patients in this cohort, 365 had died and 92 were lost to follow-up during this period. The deceased cohort was older than those lost to the follow-up, which is reasonable because age is the most significant risk factor for death. There was no significant difference between the two cohorts in the other baseline characteristics (Supplementary Table S2). Survival curves showed that both a low CXI and low CXI were significantly associated with worse OS and DFS (Figures 1A–D). When stratified by different TNM stages, a low CXI and low H-CXI were significantly associated with worse OS and DFS in patients with TNM stage II or stage III (Supplementary Figures S2, S3). The univariate analysis showed that age ≥ 75 years, a low CXI, a low H-CXI, cachexia, ASA grade III, low differentiation of tumor, and TNM stage III were associated with worse OS. The multivariate analysis including a low CXI showed that age ≥ 75 years (*p* < 0.001), low CXI (*p* = 0.001), ASA grade III (*p* = 0.004), low tumor differentiation (*p* = 0.004), and TNM stage III (*p* = 0.032) were independent prognostic factors for OS. When a low H-CXI was included in the multivariate model instead of a low CXI, the former remained a significant risk factor (*p* = 0.007). Other independent risk factors remained the same in both multivariate models (Table 4). The univariate analysis for DFS showed that age ≥ 75 years, a low CXI, a low H-CXI, cachexia, NRS2002 ≥ 3, ASA grade III, low differentiation of tumor, and TNM stage III were associated with worse DFS, whereas laparoscopic surgery was associated with better DFS. The multivariate analysis including a low CXI showed that age ≥ 75 years (*p* < 0.001), a low CXI (*p* = 0.001), ASA grade III (*p* = 0.021), low tumor differentiation (*p* = 0.003), and TNM stage III (*p* < 0.001) were independent prognostic factors for DFS. When a low H-CXI was included in the multivariate model instead of a low CXI, a low H-CXI remained a significant prognostic factor (*p* < 0.001) for DFS (Table 5).

**Table 4:** Univariate and multivariate analyses of factors associated with overall survival^#^.

Factors	Univariate analysis		Multivariate analysis			
			Low CXI		Low H-CXI	
	HR (95%CI)	*p*	HR (95%CI)	*p*	HR (95%CI)	*p*
Age, years						
≥75/<75	1.741 (1.393–2.177)	**<0.001** ^*^	1.545 (1.218–1.959)	**< 0.001** ^*^	1.543 (1.215–1.960)	**< 0.001** ^*^
Sex						
Male/Female	0.963 (0.780–1.187)	0.721	0.987 (0.800–1.219)	0.906	0.991 (0.803–1.224)	0.935
BMI, kg/m^2^						
≥25/<25	0.982 (0.770–1.252)	0.883	–	–	–	–
Low CXI						
Yes/No	1.542 (1.237–1.922)	**<0.001** ^*^	1.476 (1.178–1.849)	**0.001** ^*^	–	–
Low H-CXI						
Yes/No	1.534 (1.230–1.913)	**<0.001** ^*^	–	–	1.369 (1.090–1.720)	**0.007** ^*^
Cachexia						
Yes/No	1.308 (1.052–1.626)	**0.016** ^*^	1.187 (0.915–1.540)	0.197	1.212 (0.936–1.570)	0.145
Anemia						
Yes/No	1.100 (0.889–1.360)	0.382	–	–	–	–
NRS2002						
≥3/<3	1.231 (0.995–1.523)	0.055	0.968 (0.748–1.254)	0.807	0.958 (0.741–1.239)	0.743
ASA grade						
III/I, II	1.688 (1.264–2.254)	**<0.001** ^*^	1.574 (1.168–2.121)	**0.003** ^*^	1.480 (1.100–1.992)	**0.010** ^*^
Previous abdominal surgery						
Yes/No	0.998 (0.768–1.297)	0.989	–	–	–	–
Tumor location						
Rectum/Colon	0.986 (0.800–1.216)	0.895	–	–	–	–
Differentiation of tumor						
Low/median or high	1.446 (1.108–1.889)	**0.007** ^*^	1.362 (1.036–1.791)	**0.027** ^*^	1.325 (1.008–1.742)	**0.044** ^*^
TNM stage						
II/<I	1.011 (0.764–1.337)	0.940	0.938 (0.708–1.244)	0.656	0.943 (0.711–1.249)	0.680
III/<I	1.479 (1.127–1.943)	**0.005** ^*^	1.332 (1.007–1.763)	**0.045** ^*^	1.345 (1.017–1.779)	**0.038** ^*^
Combined organ resection						
Yes/No	0.829 (0.523–1.316)	0.427	–	–	–	–
Laparoscopic surgery						
Yes/No	0.838 (0.682–1.031)	0.094	0.943 (0.763–1.165)	0.585	0.952 (0.770–1.176)	0.648

HR, hazard ratio; CI, confidence interval; BMI, body mass index; CXI, cachexia index; NRS2002, Nutritional Risk Screening 2002; ASA, American Society of Anesthesiologists; TNM, tumor-node-metastasis. ^*^ Statistically significant. ^#^ Cox proportional hazards models were utilized.

**Table 5:** Univariate and multivariate analyses of factors associated with disease-free survival^#^.

Factors	Univariate analysis		Multivariate analysis			
			Low CXI		Low H-CXI	
	HR (95%CI)	*p*	HR (95%CI)	*p*	HR (95%CI)	*p*
Age, years						
≥75/<75	1.933 (1.606–2.327)	**<0.001** ^*^	1.756 (1.437–2.145)	**<0.001** ^*^	1.710 (1.398–2.092)	**<0.001** ^*^
Sex						
Male/Female	0.906 (0.760–1.081)	0.275	0.942 (0.788–1.126)	0.511	0.939 (0.786–1.122)	0.489
BMI, kg/m^2^						
≥25/<25	0.917 (0.744–1.130)	0.417	–	–	–	–
Low CXI						
Yes/No	1.705 (1.419–2.050)	**<0.001** ^*^	1.611 (1.334–1.946)	**<0.001** ^*^		
Low H-CXI						
Yes/No	1.838 (1.532–2.205)	**<0.001** ^*^	–	–	1.642 (1.357–1.986)	**<0.001** ^*^
Cachexia						
Yes/No	1.277 (1.060–1.539)	**0.010** ^*^	1.114 (0.891–1.392)	0.345	1.148 (0.920–1.431)	0.222
Anemia						
Yes/No	1.166 (0.974–1.394)	0.094	0.901 (0.745–1.089)	0.280	0.876 (0.723–1.062)	0.179
NRS2002						
≥3/<3	1.227 (1.024–1.471)	**0.026** ^*^	0.980 (0.787–1.220)	0.855	0.967 (0.778–1.201)	0.759
ASA grade						
III/I, II	1.478 (1.144–1.908)	**0.003** ^*^	1.376 (1.056–1.792)	**0.018** ^*^	1.269 (0.975–1.651)	0.076
Previous abdominal surgery						
Yes/No	1.132 (0.914–1.401)	0.257	–	–	–	–
Tumor location						
Rectum/Colon	0.958 (0.802–1.145)	0.640	–	–	–	–
Differentiation of tumor						
Low/median or high	1.529 (1.222–1.913)	**<0.001** ^*^	1.417 (1.124–1.785)	**0.003** ^*^	1.377 (1.093–1.735)	**0.007** ^*^
TNM stage						
II/<I	1.223 (0.960–1.557)	0.103	1.142 (0.894–1.458)	0.287	1.151 (0.902–1.470)	0.258
III/<I	1.772 (1.397–2.248)	**<0.001** ^*^	1.630 (1.277–2.082)	**<0.001** ^*^	1.646 (1.290–2.101)	**<0.001** ^*^
Combined organ resection						
Yes/No	1.076 (0.756–1.531)	0.684	–	–	–	–
Laparoscopic surgery						
Yes/No	0.839 (0.705–0.999)	**0.049** ^*^	0.938 (0.784–1.121)	0.480	0.951 (0.796–1.138)	0.584

HR, hazard ratio; CI, confidence interval; BMI, body mass index; CXI, cachexia index; NRS2002, Nutritional Risk Screening 2002; ASA, American Society of Anesthesiologists; TNM, tumor-node-metastasis. ^*^ Statistically significant. ^#^ Cox proportional hazards models were utilized.

### 3.4 Cancer cachexia

Cancer cachexia was identified in 361 (25.6%) of all the patients using Fearon’s criteria. Patients with cachexia had a lower CXI (*p* < 0.001) and lower H-CXI (*p* < 0.001) than those without cachexia. Moreover, a higher TNM stage was associated with a lower CXI (*p* = 0.015) and lower H-CXI (*p* = 0.006). The univariate analysis showed that both a low CXI (*p* = 0.001) and low H-CXI (*p* = 0.039) were associated with cancer cachexia (Table 6). The multivariate analysis showed that a low CXI was an independent risk factor for cachexia (*p* = 0.024), whereas a low H-CXI cannot independently predict cachexia after adjusting for the same covariates (*p* = 0.944). Survival curves showed a worse OS and DFS in patients with cancer cachexia than those without cachexia (Figures 1E,F). Cancer cachexia was associated with worse OS and DFS in the univariate analyses but did not remain a significant risk factor in the multivariate analyses (Tables 4, 5). Moreover, no significant association was found between cancer cachexia and postoperative complications (*p* = 0.192) (Table 2).

**Table 6:** Univariate and multivariate analyses of factors associated with cancer cachexia.

Factors	Univariate analysis		Multivariate analysis			
			Low CXI		Low H-CXI	
	OR (95%CI)	*p*	OR (95%CI)	*p*	OR (95%CI)	*p*
Age, years						
≥75/<75	1.572 (1.197–2.064)	**0.001** ^*^	0.753 (0.534–1.061)	0.105	0.795 (0.564–1.122)	0.192
Sex						
Male/Female	0.815 (0.639–1.040)	0.100	0.900 (0.674–1.201)	0.474	0.896 (0.672–1.195)	0.454
BMI, kg/m^2^						
≥25/<25	0.396 (0.282–0.555)	**< 0.001** ^*^	0.529 (0.360–0.775)	**0.001** ^*^	0.509 (0.347–0.746)	**0.001** ^*^
Low CXI						
Yes/No	1.595 (1.224–2.078)	**0.001** ^*^	1.448 (1.049–1.998)	**0.024** ^*^	–	–
Low H-CXI						
Yes/No	1.326 (1.014–1.734)	**0.039** ^*^	–	–	1.012 (0.729–1.405)	0.944
Anemia						
Yes/No	1.891 (1.479–2.416)	**< 0.001** ^*^	1.439 (1.066–1.942)	**0.017** ^*^	1.481 (1.098–1.999)	**0.010** ^*^
NRS2002						
≥3/<3	11.476 (8.699–15.139)	**< 0.001** ^*^	11.394 (8.487–15.297)	**< 0.001** ^*^	11.287 (8.416–15.137)	**< 0.001** ^*^
ASA grade						
III/I, II	1.052 (0.720–1.536)	0.793	–	–	–	–
Previous abdominal surgery						
Yes/No	1.201 (0.894–1.613)	0.224	–	–	–	–
Tumor location						
Rectum/Colon	0.760 (0.592–0.976)	**0.032** ^*^	1.045 (0.774–1.411)	0.775	1.054 (0.780–1.422)	0.733
Differentiation of tumor						
Low/median or high	1.305 (0.942–1.806)	0.109	–	–	–	–
TNM stage						
II/<I	1.681 (1.202–2.351)	**0.002** ^*^	1.467 (0.994–2.166)	0.054	1.468 (0.955–2.164)	0.053
III/<I	2.230 (1.592–3.123)	**< 0.001** ^*^	2.120 (1.430–3.142)	**< 0.001** ^*^	2.131 (1.439–3.154)	**< 0.001** ^*^

OR, odds ratio; CI, confidence interval; BMI, body mass index; CXI, cachexia index; NRS2002, Nutritional Risk Screening 2002; ASA, American Society of Anesthesiologists; TNM, tumor-node-metastasis. ^*^ Statistically significant.

## 4 Discussion

### 4.1 H-CXI vs. CXI for detecting baseline characteristics and cancer cachexia

The present study showed that both a low CXI and low H-CXI were associated with older age, a lower BMI, SMI, and HGS, lower levels of albumin and hemoglobin, and higher NLR, which indicated that both the CXI and H-CXI reflect body nutritional and functional status as well as systemic inflammation. However, a low CXI but not a low H-CXI was identified as an independent risk factor for cancer cachexia in the present study. Low skeletal muscle mass was one of the diagnostic criteria for cancer cachexia according to the international consensus, whereas HGS was not included in the current diagnostic criteria for cancer cachexia (1). The skeletal muscle index is a parameter in the calculation of CXI (12), which can explain the better predictive value of the CXI for cancer cachexia over the H-CXI. Our results indicated that HGS cannot fully substitute SMI in the detection of cancer cachexia. The measurement of skeletal muscle mass is still imperative for an accurate assessment of cachexia state based on the current diagnostic criteria for cancer cachexia.

### 4.2 The H-CXI and CXI rather than cancer cachexia predicted clinical outcomes

Several previous studies have investigated the influence of cancer cachexia on postoperative outcomes in colorectal cancer, and the results were inconsistent (7, 10, 20). The present study showed that the CXI and H-CXI rather than cancer cachexia predicted worse postoperative outcomes. Our finding was consistent with a previous study, which showed that a low CXI instead of cachexia was associated with a decreased OS after surgery for colorectal cancer (15). The diagnosis of cancer cachexia requires the estimation of weight loss, which might be influenced by the subjectivity of patients’ recall. On the contrary, the CXI and H-CXI were calculated based on objective parameters such as SMI, HGS, and the results of hematological examinations. The SMI and HGS were key elements for the diagnosis of sarcopenia, serum albumin reflected the nutritional status, and higher NLR indicated higher levels of systemic inflammation (12). Moreover, these three factors were closely correlated with each other. The chronic inflammatory state was the most important feature shared by sarcopenia and malnutrition (21). Both malnutrition and systemic inflammation played a key role in the development of sarcopenia (22). Sarcopenia (18), nutritional status (23), and systemic inflammation (24) were all associated with worse prognosis in patients with cancer. The CXI and H-CXI incorporated all three factors, which can explain their superior prognostic value for postoperative outcomes over cancer cachexia. Previous studies have indicated that current pharmacologic agents used in cancer cachexia may improve weight but not the prognosis (25, 26). The superior prognostic value of the CXI and H-CXI over Fearon’s criteria for cancer cachexia recommended we recognize cachexia beyond weight loss alone, focusing more on muscle mass, physical function, and inflammatory state (25).

### 4.3 H-CXI vs. CXI for the prognostic value

The present study showed that both a low CXI and low H-CXI were independently associated with worse OS and DFS after surgery. Moreover, patients with a higher TNM stage had a lower CXI and lower H-CXI, indicating that both the CXI and H-CXI were good indicators for tumor progression in colorectal cancer. Notably, a low H-CXI but not a low CXI was identified as an independent risk factor for postoperative complications in the present study. Moreover, a low H-CXI but not a low CXI was associated with a longer hospital stay after surgery and costs. This finding indicated that the H-CXI was a better predictive factor for short-term postoperative outcomes than the CXI. Hand-grip strength is the most common measure for the assessment of muscle function (27), which has been proven to be an appropriate proxy for muscle status in many situations (27, 28). Some previous studies have demonstrated the superior prognostic value of HGS over skeletal muscle mass for the prediction of clinical outcomes (29, 30). The procedure for the measurement of the skeletal muscle index by CT scans is relatively complicated, requiring experienced technicians to determine the border of muscle mass with the assistance of specialized image processing software. The measurement of the skeletal muscle index is not a routine clinical practice in most medical centers, except for research purposes. The skeletal muscle index is not included in the formal report of CT examinations in routine clinical work. Moreover, abdominal CT scans were not routinely performed in many other types of cancers, except for gastrointestinal cancers. All the abovementioned factors impede the generalization of the skeletal muscle index in clinical practice. On the contrary, hand-grip strength can be easily measured in various types of patients. The results of hand-grip strength tests can be immediately acquired to promote rapid preoperative assessment and risk stratification. Therefore, the H-CXI is the most readily usable measure among the above three tools. Our study indicated that the H-CXI is a superior biomarker over the original CXI in terms of its better prognostic value for short-term postoperative outcomes and its convenience in clinical applications.

### 4.4 Advantages and limitations

Our present study was the first to compare the prognostic value between the CXI and H-CXI. The advantages of our study are the large sample size and the prospective data collection. However, the single-center study design may impede the generalization of our conclusion, which is a limitation of the present study. In addition, we determined the cutoff value for the cachexia index based on the sex-specific lower quartile. Therefore, our cutoff values may not be feasible for other studies, which is also a limitation of our study. However, there was no consensus on the optimal cutoff for the cachexia index. Previous studies set the cutoff values either by the receiver operating characteristic (ROC) curves (13, 15) or by the median CXI of men and women, respectively (31). Since the present study aimed to compare the prognostic value between the CXI and H-CXI, the same proportion of patients (1/4) below the cutoff values could make a rational comparison.

## 5 Conclusion

In conclusion, the present study showed that the CXI and H-CXI exhibited better prognostic value than cancer cachexia for the prediction of postoperative outcomes in patients who underwent radical colectomy for colorectal cancer. The H-CXI had a similar predictive value for long-term survival and a better predictive value for short-term postoperative outcomes than the original CXI, whereas the CXI had a closer correlation with Fearon’s criteria for cancer cachexia. Ideal tools for the assessment of cancer cachexia should incorporate not only weight loss but also muscle mass, physical function, and inflammatory state.

## Data availability statement

The original contributions presented in the study are included in the article/Supplementary material, further inquiries can be directed to the corresponding authors.

## Ethics statement

The studies involving humans were approved by The Ethical Review Board of The First Affiliated Hospital of Wenzhou Medical University. The studies were conducted in accordance with the local legislation and institutional requirements. The participants provided their written informed consent to participate in this study.

## Author contributions

X-LY: Writing – original draft, Writing – review & editing, Project administration, Data curation, Formal analysis. L-MW: Writing – review & editing, Formal analysis, Data curation. X-BT: Data curation, Writing – original draft, Writing – review & editing. Z-ZL: Writing – review & editing, Data curation, Formal analysis. ZZ: Data curation, Writing – review & editing, Formal analysis. H-JJ: Writing – review & editing, Data curation. Z-TC: Writing – review & editing, Data curation. D-HC: Writing – review & editing, Data curation. J-YL: Writing – review & editing, Data curation. XS: Conceptualization, Writing – review & editing. D-DH: Conceptualization, Writing – review & editing.
