# Supplementary material for: Comparison of the cachexia index based on hand-grip strength (H-CXI) with the original CXI for the prediction of cancer cachexia and prognosis in patients who underwent radical colectomy for colorectal cancer
Source: Front Nutr. 2024 Feb 20;11:1290299. doi: 10.3389/fnut.2024.1290299 (PMC10912503; doi:10.3389/fnut.2024.1290299)
Supplement: Supplementary file 1 [file Data_Sheet_1.DOCX]

Supplementary Material

**
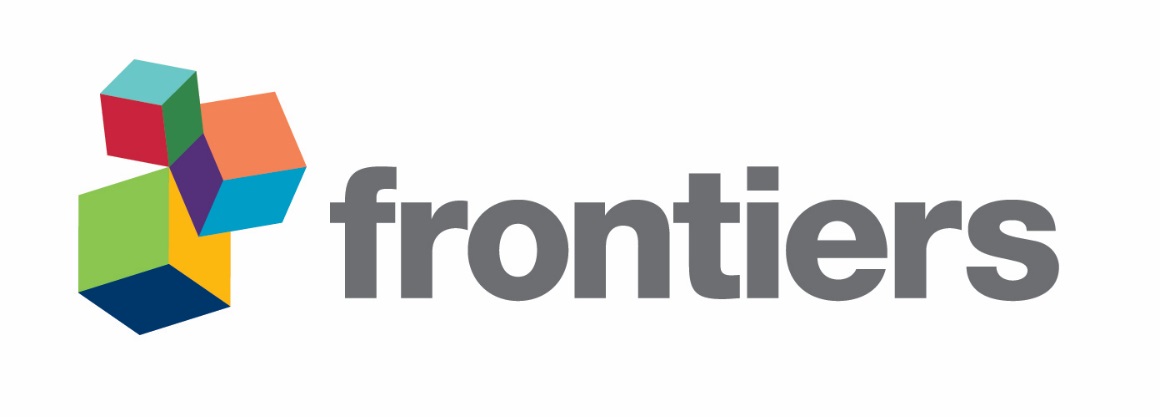
**

## Supplementary Figures

**Supplementary Figure 1.** Representative images of patients without (A) and with (B) skeletal muscle atrophy.


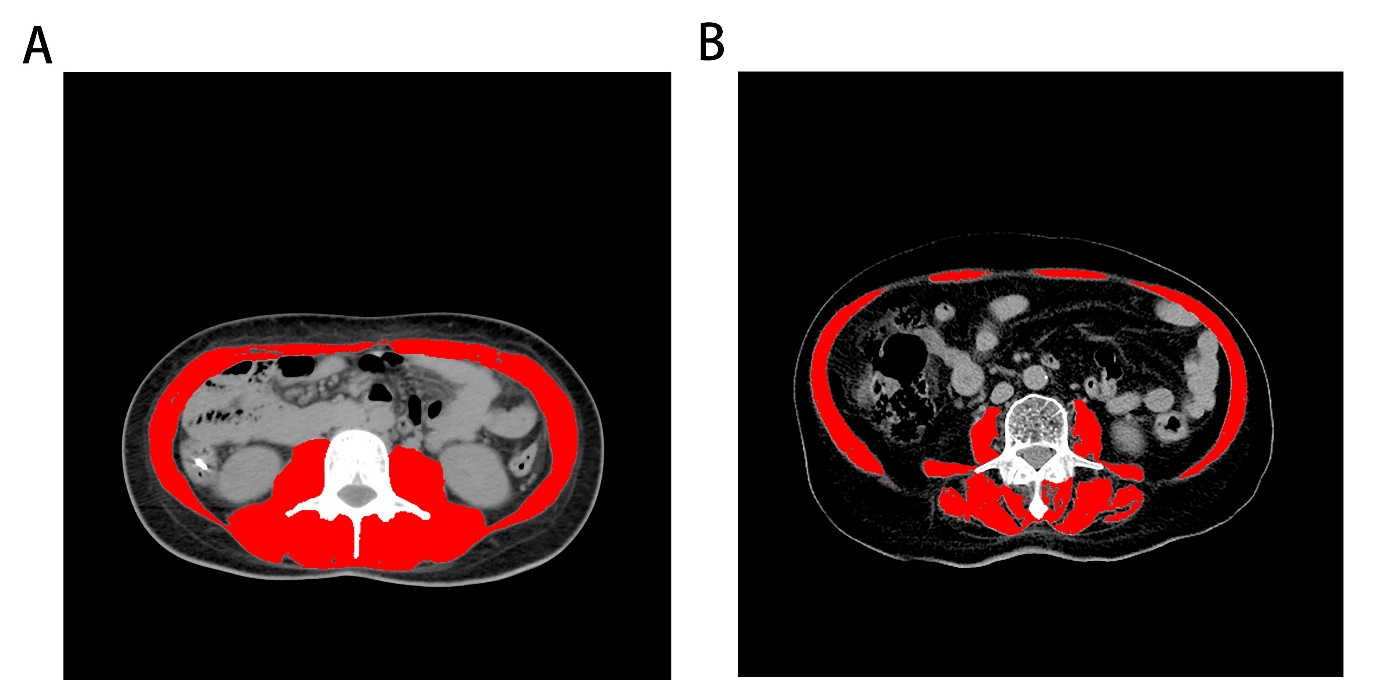


**Supplementary Figure 2.** Kaplan Meier survival curves for associations between overall survival and low CXI or low H-CXI stratified by different TNM stages.

Overall survival of patients with low CXI and high CXI in (A) TNM stage I, (B) stage II, and (C) stage III.

Overall survival of patients with low H-CXI and high H-CXI in (D) TNM stage I, (E) stage II, and (F) stage III.


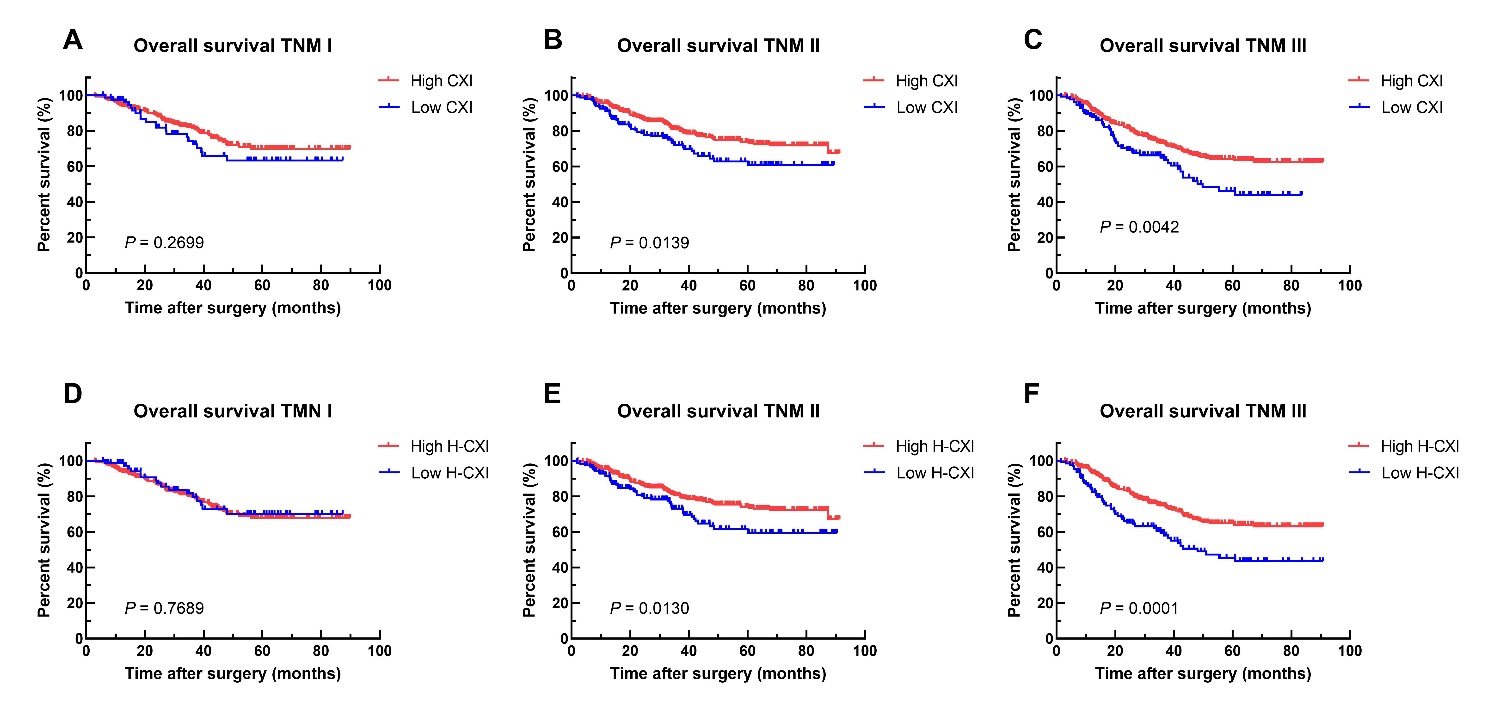


**Supplementary Figure 3.** Kaplan Meier survival curves for associations between disease-free survival and low CXI or low H-CXI stratified by different TNM stages.

Disease-free survival of patients with low CXI and high CXI in (A) TNM stage I, (B) stage II, and (C) stage III.

Disease-free survival of patients with low H-CXI and high H-CXI in (D) TNM stage I, (E) stage II, and (F) stage III.


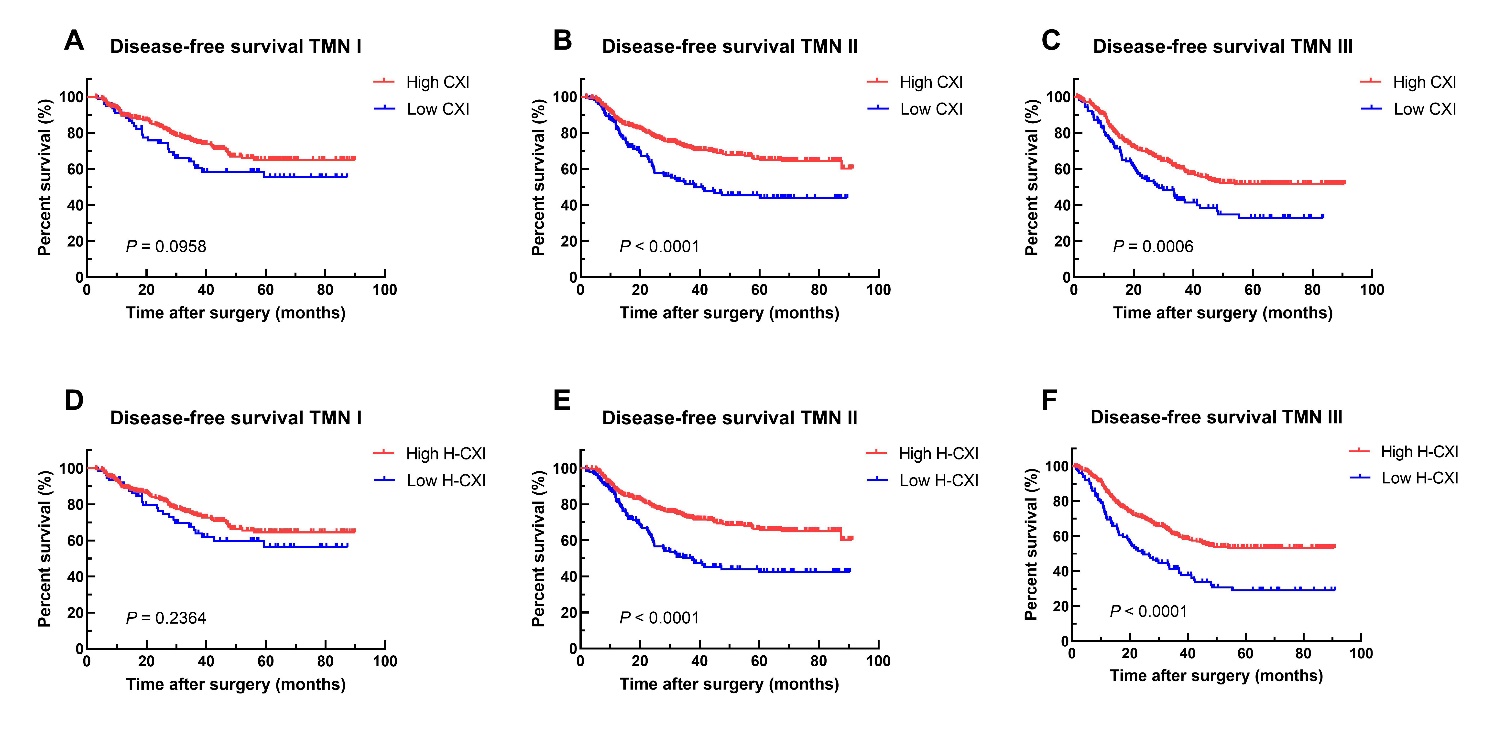


## Supplementary Tables

**Supplementary Table 1.** Details of short-term postoperative outcomes

|  | Total  (n = 1411) | High CXI  (n =1058) | Low CXI  (n =353) | P | High H-CXI  (n =1058) | Low H-CXI  (n =353) | P |
| --- | --- | --- | --- | --- | --- | --- | --- |
| **Total complications** † | 347 (24.6) | 241(22.8) | 106 (30.0) | 0.006^*^ | 235(22.2) | 112 (31.7) | <0.001* |
| Anastomotic leakage | 48 | 35 | 13 | 0.737 | 35 | 13 | 0.737 |
| Arrhythmia | 4 | 1 | 3 | 0.083 | 2 | 2 | 0.564 |
| Delayed gastric emptying | 5 | 3 | 2 | 0.797 | 4 | 1 | 0.797 |
| Duodenal fistula | 1 | 0 | 1 | 0.564 | 1 | 0 | 0.564 |
| Delirium | 2 | 2 | 0 | 1.000 | 1 | 1 | 1.000 |
| Gastrointestinal hemorrhage | 2 | 0 | 2 | 0.102 | 1 | 1 | 1.000 |
| Heart failure | 4 | 4 | 0 | 0.563 | 4 | 0 | 0.563 |
| Ileus | 28 | 17 | 11 | 0.078 | 16 | 12 | 0.028^*^ |
| Intestinal infection | 3 | 3 | 0 | 0.738 | 3 | 0 | 0.738 |
| Intra-abdominal infection | 100 | 75 | 25 | 0.997 | 69 | 31 | 0.152 |
| Intra-abdominal hemorrhage | 16 | 14 | 2 | 0.383 | 11 | 5 | 0.773 |
| Hepatic failure | 1 | 0 | 1 | 0.564 | 0 | 1 | 0.564 |
| Lymphatic fistula | 7 | 5 | 2 | 0.826 | 6 | 1 | 0.826 |
| Pancreatic fistula | 2 | 2 | 0 | 1.000 | 2 | 0 | 1.000 |
| Pleural effusion | 10 | 7 | 3 | 0.999 | 7 | 3 | 0.999 |
| Pneumonia | 27 | 19 | 8 | 0.576 | 20 | 7 | 0.912 |
| Pulmonary atelectasis | 2 | 1 | 1 | 1.000 | 1 | 1 | 1.000 |
| Pulmonary embolism | 3 | 2 | 1 | 0.738 | 2 | 1 | 0.738 |
| Respiratory failure | 2 | 2 | 0 | 1.000 | 2 | 0 | 1.000 |
| Seroperitoneum | 9 | 9 | 0 | 0.176 | 6 | 3 | 0.848 |
| Sepsis | 6 | 5 | 1 | 0.999 | 5 | 1 | 0.999 |
| Septic shock | 1 | 0 | 1 | 0.564 | 0 | 1 | 0.564 |
| Small bowel obstruction | 3 | 2 | 1 | 0.738 | 1 | 2 | 0.317 |
| Urinary system infection | 8 | 7 | 1 | 0.681 | 7 | 1 | 0.681 |
| Venous thrombosis | 38 | 22 | 16 | 0.014^*^ | 21 | 17 | 0.004^*^ |
| Wound infection | 93 | 64 | 29 | 0.156 | 65 | 28 | 0.241 |
| Hypoproteinemia | 46 | 30 | 16 | 0.120 | 28 | 18 | 0.025^*^ |
| Anemia | 4 | 2 | 2 | 0.564 | 2 | 2 | 0.564 |
| **Severe complications** ‡ | 71 | 51 | 20 | 0.529 | 49 | 22 | 0.233 |
| **Length of postoperative stay, median (IQR), days** | 12 (5) | 12 (5) | 12 (6) | 0.240 | 12 (6) | 13 (6) | 0.003^*^ |
| **Costs, median (IQR), RMB** | 51791.0  (18975.9) | 51206.8  (19036.6) | 53164.2 (19152.4) | 0.061 | 51173.4  (18949.8) | 53552.2 (19800.3) | 0.013^*^ |

CXI, cachexia index; IQR, interquartile range.

The values in the table were number of patients and percentage unless indicated otherwise.

^*^Statistically significant.

† Complications classified as grade II and above.

‡ Complications classified as grade III and above.

**Supplementary Table 2. Comparison of** **baseline characteristics between patients who had lost of follow-up and patients who had died**

| Characteristics | All (n = 457) | Died  (n = 365) | Loss of follow up  (n = 92) | P |
| --- | --- | --- | --- | --- |
| Age, median (IQR), years | 68 (17) | 69 (16) | 63 (17) | <0.001* |
| Gender |  |  |  | 0.159 |
| Female | 189 (41.4) | 145 (39.7) | 44 (47.8) |  |
| Male | 268 (58.6) | 220 (60.3) | 48 (52.5) |  |
| BMI, median (IQR), kg/m^2^ | 22.6 (4.2) | 22.5 (4.2) | 22.9 (3.7) | 0.314 |
| Weight loss |  |  |  |  |
| <2% | 298 (65.2) | 243 (66.6) | 55 (59.8) | 0.222 |
| ≥2% | 159 (34.8) | 122 (33.4) | 37 (40.2) |  |
| Albumin, median (IQR), g/L | 37.6 (6.5) | 37.5 (6.3) | 37.7 (6.6) | 0.529 |
| Hemoglobin, median (IQR), g/L | 120 (29) | 121 (29) | 118 (27) | 0.399 |
| NLR, median (IQR) | 2.66 (1.95) | 2.57 (1.95) | 2.92 (1.85) | 0.394 |
| NRS2002 scores |  |  |  | 0.050 |
| <3 | 298 (65.2) | 230 (63.0) | 68 (73.9) |  |
| ≥3 | 159 (34.8) | 135 (7.0) | 24 (26.1) |  |
| ASA grade |  |  |  | 0.377 |
| I | 125 (27.4) | 101 (27.7) | 24 (26.1) |  |
| II | 269 (58.9) | 210 (57.5) | 59 (64.1) |  |
| III | 63 (13.8) | 54 (14.8) | 9 (9.8) |  |
| Previous abdominal surgery |  |  |  | 0.079 |
| No | 363 (79.4) | 296 (81.1) | 67 (72.8) |  |
| Yes | 74 (20.6) | 69 (18.9) | 25 (27.2) |  |
| SMI, median (IQR), cm^2^/m^2^ | 42.1 (12.6) | 41.8 (13.0) | 42.6 (11.5) | 0.442 |
| HGS, median (IQR), kg | 24.0 (13.6) | 24.0 (13.1) | 23.7 (13.8) | 0.572 |
| Tumor location |  |  |  | 0.091 |
| Colon | 283 (61.9) | 219 (60.0) | 64 (69.6) |  |
| Rectum | 174 (38.1) | 146 (40.0) | 28 (30.4) |  |
| Differentiation of tumor |  |  |  | 0.743 |
| Poorly differentiated | 84 (18.4) | 299 (81.9) | 74 (80.4) |  |
| Well differentiated | 373 (81.6) | 66 (18.1) | 18 (19.6) |  |
| TNM stage |  |  |  | 0.544 |
| I | 100 (21.9) | 78 (21.4) | 22 (23.9) |  |
| II | 170 (37.2) | 133 (36.4) | 37 (40.2) |  |
| III | 187 (40.9) | 154 (42.2) | 33 (35.9) |  |
| Laparoscopy-assisted surgery |  |  |  | 0.198 |
| No | 236 (51.6) | 194 (53.2) | 42 (45.7) |  |
| Yes | 221 (48.4) | 171 (46.8) | 50 (54.3) |  |
| Combined organ resection |  |  |  | 0.862 |
| No | 433 (94.7) | 346 (94.8) | 87 (94.6) |  |
| Yes | 24 (5.3) | 19 (5.2) | 5（5.4) |  |

IQR, interquartile range; BMI, body mass index; NLR, neutrophil to lymphocyte ratio; NRS2002, Nutritional Risk Screening 2002; ASA, American Society of Anaesthesiologists; SMI, skeletal muscle index; HGS, hand-grip strength; TNM, tumor–node–metastasis.

The values in the table were number of patients and percent unless indicated otherwise.

**^*^**Statistically significant.

## 
